
JOURNAL INFORMATION
==============================
NLM Title Abbreviation: ScientificWorldJournal
Journal ID: ScientificWorldJournal
NLM Journal ID: 101131163
Journal ID: TSWJ

The Scientific World Journal

ISSN: 2356-6140
EISSN: 1537-744X
Publisher: Wiley

ARTICLE INFORMATION
==============================
PMCID: PMC5849132
PMID: 19112536
DOI: 10.1100/tsw.2008.162
Article ID: 270589
Article version: 1
Subjects: Clinical Image

An Unusual Presentation: Renal Tuberculosis

Gurski Jennifer L. *
Baker Karen C.
Department of Surgery, Urology Service, Madigan Army Medical Center, Tacoma, WA, USA
*Jennifer L. Gurski: jennifer.gurski@amedd.army.milAcademic Editor: Daniel Batlle

Publication date: 2008
Electronic publication date: 2008 Dec 25
Volume: 8
First page: 1254
Last page: 1255
Received 2008 Nov 26; Revised 2008 Dec 9; Accepted 2008 Dec 16
Copyright: Copyright © 2008 Jennifer L. Gurski and Karen C Baker.
Copyright year: 2008
License: This is an open access article distributed under the Creative Commons Attribution License, which permits unrestricted use, distribution, and reproduction in any medium, provided the original work is properly cited.
License URL: https://creativecommons.org/licenses/by/3.0/

Keywords: tuberculosis, extrapulmonary, diagnosis, imaging

==============================
Clinical Image
*TheScientificWorldJOURNAL* (2008) 8, 1254–1255
ISSN 1537-744X; DOI 10.1100/tsw.2008.162

TheScientificWorldJOURNAL
www.thescientificworld.com

### An Unusual Presentation: Renal Tuberculosis

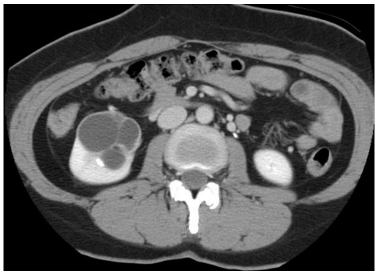

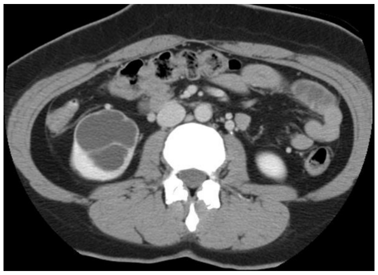

**FIGURE 1A and B.** Contrast CT images demonstrating a 4.5- × 4.5-cm cystic mass with multiple internal enhancing septations at the inferior pole of the right kidney. Contrast is visible in the collecting system in Fig. 1A.

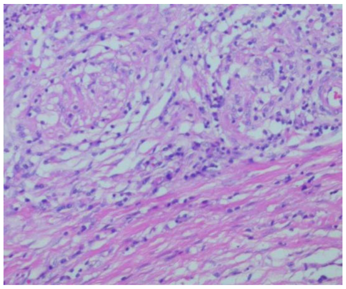

**FIGURE 2.** Nephrectomy specimen showing chronic interstitial nephritis. Hematoxylin-eosin stain, original magnification ×100.

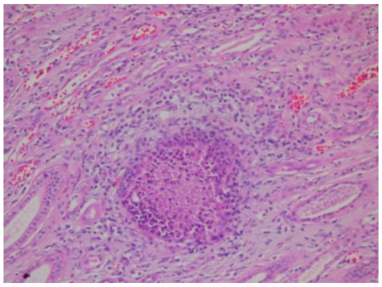

**FIGURE 3.** Caseating granulomatous renal tuberculosis. Epithelioid cells surround a central area of necrosis that appears irregular, amorphous, and pink. Hematoxylin-eosin stain, original magnification ×100.

Jennifer L. Gurski* and Karen C. Baker

*Department of Surgery, Urology Service, Madigan Army Medical Center, Tacoma, WA*

**E-mail**: Jennifer.gurski@amedd.army.mil, Karen.c.baker@us.army.mil

*Received November 26, 2008; Revised December 9, 2008; Accepted December 16, 2008; Published December 25, 2008*

**KEYWORDS**: tuberculosis, extrapulmonary, diagnosis, imaging

*Corresponding author.
©2008 with author.
Published by TheScientificWorld; www.thescientificworld.com

A 44-year-old Philippine woman with a history of positive purified protein derivative (PPD) was referred for hydronephrosis that had been discovered on an ultrasound performed for a single, fleeting episode of right-sided flank pain. The CT scan demonstrated a well-circumscribed cystic mass with enhancing septations at the inferior pole of the right kidney (Fig. 1). The differential diagnosis included xanthogranulomatous pyelonephritis and abscess, although both were thought to be unlikely in the absence of a suggestive history or signs of infection. Given the patient's age and radiographic enhancement, the lesion appeared concerning for multiloculated cystic nephroma vs. cystic renal cell carcinoma. After counseling her as to her options, she underwent right laparoscopic nephrectomy. The pathologic examination revealed caseating, granulomatous, interstitial nephritis (Figs. 2 and 3). Acid-fast organisms were present on the AFB stain.

This case represents an unusual radiographic appearance of renal tuberculosis (TB). Genitourinary TB is the second most common form of extrapulmonary TB after peripheral lymphadenopathy[1]. The manifestations of renal TB include hematuria, sterile pyuria, colic, and renal failure. Constitutional symptoms, such as fever, weight loss, and fatigue, are less common. Patients may be asymptomatic; however, those with renal TB will usually have evidence of concomitant inactive pulmonary disease.

Radiographic findings of early renal TB often are nonspecific and of limited diagnostic value. The calyces may have a "moth eaten" appearance secondary to papillary necrosis. In the later stages of TB, ureteral and infundibular strictures, "beading" hydronephrosis, cavitation, and focal calcification may be present[2]. A small, calcified, nonfunctioning renal unit (the so-called "putty kidney") representing autonephrectomy may be visualized[3].

Renal TB should be included in the differential diagnosis of renal lesions in patients with the appropriate exposure and/or travel history, or who are PPD positive.

### REFERENCES

1. Wise, G.J. and Marella, V.K. (2003) Genitourinary manifestations of tuberculosis. *Urol. Clin. North Am.* **30,** 111–121.
2. Mandell, G.L., Douglas, R.G., Bennett, J.E., and Dolin, R. (2005) *Mandell, Douglas, and Bennett's Principles and Practice of Infectious Diseases*. Elsevier Churchill Livingstone, Philadelphia.
3. Kocakoc, E., Bhatt, S., and Dogra, V.S. (2005) Renal multidector row CT. *Radiol. Clin. North Am.* **43,** 1021–1047.

**This article should be cited as follows:**

Gurski, J.L. and Baker, K.C. (2008) An unusual presentation: renal tuberculosis. *TheScientificWorldJOURNAL* **8**, 1254–1255. DOI 10.1100/tsw.2008.162.
